# Development of pig welfare assessment protocol integrating animal-, environment-, and management-based measures

**DOI:** 10.1186/s40781-014-0034-0

**Published:** 2015-01-09

**Authors:** Anriansyah Renggaman, Hong L Choi, Sartika IA Sudiarto, Laura Alasaarela, Ok S Nam

**Affiliations:** Department of Agriculture Biotechnology and Research Institute for Agriculture and Life Sciences, Seoul National University, Seoul, Republic of Korea; College of Veterinary Medicine, Seoul National University, Seoul, Republic of Korea; Faculty of Veterinary Medicine, University of Helsinki, Helsinki, Finland

**Keywords:** Animal welfare, Environment-based measure, Pig farming, Intensive farming system, Welfare assessment protocol

## Abstract

**Background:**

Due to increased interest in animal welfare, there is now a need for a comprehensive assessment protocol to be used in intensive pig farming systems. There are two current welfare assessment protocols for pigs: Welfare Quality® Assessment Protocols (applicable in the Europe Union), that mostly focuses on animal-based measures, and the Swine Welfare Assurance Program (applicable in the United States), that mostly focuses on management- and environment-based measures. In certain cases, however, animal-based measures might not be adequate for properly assessing pig welfare status. Similarly, welfare assessment that relies only on environment- and management-based measures might not represent the actual welfare status of pigs. Therefore, the objective of this paper was to develop a new welfare protocol by integrating animal-, environment-, and management-based measures. The background for selection of certain welfare criteria and modification of the scoring systems from existing welfare assessment protocols are described.

**Methods:**

The developed pig welfare assessment protocol consists of 17 criteria that are related to four main principles of welfare (good feeding, good housing, good health, and appropriate behavior). Good feeding, good housing, and good health were assessed using a 3-point scale: 0 (good welfare), 1 (moderate welfare), and 2 (poor welfare). In certain cases, only a 2-point scale was used: 0 (certain condition is present) or 2 (certain condition is absent). Appropriate behavior was assessed by scan sampling of positive and negative social behaviors based on qualitative behavior assessment and human-animal relationship tests.

**Results:**

Modification of the body condition score into a 3-point scale revealed pigs with a moderate body condition (score 1). Moreover, additional criteria such as feed quality confirmed that farms had moderate (score 1) or poor feed quality (score 2), especially those farms located in a high relative humidity region.

**Conclusions:**

The developed protocol can be utilized to assess welfare status in an intensive pig farming system. Although further improvements are still needed, this study is a first step in developing a pig welfare assessment protocol that combines animal-, environment-, and management-based measures.

## Background

Animal welfare reflects the wellbeing of an animal and comprises an animal’s physical and mental health. Animal welfare is affected by environmental conditions and innate behavior [1]. In Europe, animal welfare has been used as livestock product quality certification parameter [2]. Importance of animal welfare varies among countries or regions. For example, the high awareness of European consumers regarding the welfare of livestock animals has led to livestock welfare regulations [3]. In contrast, Asian consumers show no or little interest in animal welfare, especially that of livestock animals.

Nevertheless, the development of a welfare assessment protocol in Asian countries such as Republic of Korea is needed to advise farmers in improving the welfare of their livestock. Moreover, a welfare certification scheme to standardize livestock products would assist trade between countries in the same region [4]. There are two current welfare assessment protocols for pigs, Welfare Quality® Assessment Protocols and Swine Welfare Assurance Program (SWAP). Welfare Quality® Assessment Protocols are applicable in the European Union and mostly focuses on animal-based measures [5]. Swine Welfare Assurance Program (SWAP) is applicable in the United States and mostly focuses on environment- and management-based measures [6]. These current pig welfare assessment protocols are not necessary applicable in Asian countries. The main obstacle is the difference in how livestock welfare is viewed among the different regions.

The objective of this paper was to develop a new protocol to assess welfare status of growing and fattening pigs in Asian countries such as Republic of Korea by integrating animal-, management-, and environment-based measures. In certain cases, animal-based measures might not be adequate to properly assess pig welfare status. Similarly, welfare assessment that relies only on environment- and management-based measures might not represent the actual welfare status of the pig. In developing the new protocol, the background for selection of certain welfare criteria and modification of the scoring systems from existing welfare assessment protocols are described. Moreover, the new protocol was validated by assessing the welfare status of two growing pig farms.

## Methods

### Farm sample

The developed welfare assessment protocol was tested at two experimental growing pig farms of Seoul National University, Seoul, Republic of Korea. Both farms were representative of early growing and fattening phases. Both farms were conventional indoor farms on concrete flooring with a partially-slatted floor. Pigs entered the farm with an average weight of 20 kg and were removed for slaughter at an average weight of 110 kg. The growing pigs observed in the present study were handled humanely and did not received any constraint throughout the observation. Welfare quality was assessed by two observers, each of which was responsible for assessing two main principles of animal welfare.

### Protocol for growing pigs

Similar to Welfare Quality® Assessment Protocols, the developed protocol consisted of four main principles of animal welfare: good feeding, good housing, good health, and appropriate behavior. The four main principles are subdivided into 17 independent criteria that are a combination of animal-, environment-, and management-based measures (Table 1). On the other hand, Welfare Quality® Assessment Protocols only subdivides the four main principles into 12 independent criteria and focus on animal-based measures.

**Table 1:** **Developed measures for welfare assessment of growing pigs on farms**

**Welfare criteria**	**Measures**
***I. Good feeding***	
1 Absence of prolonged hunger^1^	Body condition scores
2 Feed quality^2^	Feed condition^4^
3 Absence of prolonged thirst^1^	Water supply^4^
***II. Good housing***	
4 Environmental condition^2^	Temperature and relative humidity^5^
5 Ventilation status (air quality)^2, 3^	Particulate matter and ammonia concentration^5^
6 Comfort around resting^1^	Bursitis and manure on the body
7 Thermal comfort^1^	Shivering, panting, and huddling
8 Ease of movement^1^	Space allowance
9 Other facility condition^3^	Conditions of floor, fencing, feeder, and other facilities inside the farm^4^
***III. Good health***	
10 Absence of injuries^1^	Lameness, wounds, tail biting
11 Absence of disease^1^	Coughing, sneezing, pumping, twisted snouts, rectal prolapse, scouring, skin condition, ruptures, and hernia
12 Health management^3^	Veterinary-client-patient relationship, medical record, and hospital pen^4^
13 Euthanasia^2^	Number of euthanized animal and euthanasia methods
***IV. Appropriate behavior***	
14 Expression of social behavior^1^	Negative and positive social behavior
15 Expression of other behavior^1^	Exploratory behavior
16 Good human animal relationship^1^	Fear of humans
17 Positive emotional state^1^	Qualitative behavior assessment

^1^Based on Welfare Quality® Assessment Protocols [5].

^2^New criteria added by the authors.

^3^Based on Swine Welfare Assurance Program [6].

^4^Management-based measure.

^5^Environment-based measure.

As the first step of the farm survey, general farm information was recorded by interviewing farmers using a previously prepared questionnaire. During the interview, observation methods were explained to get farmers consent. The questionnaire recorded information on mortality rate, total number of pigs in the farm and observation house, total number of pens in the observation house, pen area, ventilation system, average weight of observed pigs, and age of observed pigs. After collecting general farm information, behavioral observations were carried out, followed by evaluation of animal- and environment-based measures related to good feeding, good housing, and good health. Farmers had given their consent prior to the observation.

### Measurement of good feeding, good housing, and good health

Measurement for these welfare principles was carried out at the pen or individual pig level using a 3-point scale: 0 for good welfare, 1 for compromised welfare, and 2 for unacceptable welfare. The number of pens or pigs that got a score of 0, 1, or 2 was recorded, and the proportion of total pens or pigs that got a score of 0, 1, or 2 were calculated. When welfare status could not be classified into one of the three different states as mentioned above, a binary scale of 0 when a certain case was absent or 2 when a certain case was present was used (Table 2).

**Table 2:** **Developed scoring scale for good feeding, housing, and health**

**Measures**	**Scores**	**Description**
Body condition	0	Animal with a good body condition
	1	Animal with moderate body condition
	2	Animal with a poor body condition (lean animals)
Feed quality^1^	0	Less than 1/3 is clod and there is no smell
	1	More than 1/3 but less than 1/2 is clod and doesn’t smell or less than 1/3 is clod but smells sour
	2	More than half is clod and smells sour
Temperature^2^	0	Temperature is appropriate for the pigs
	2	Temperature is inappropriate for the pigs
Ammonia concentration^2^	0	Ammonia concentration below 50 ppm
	2	Ammonia concentration exceeded 50 ppm
Bursitis	0	No existence of bursa
	1	One or several small bursa or one medium bursa in same leg
	2	Several medium bursa or one big bursa in same leg
Manure on the body	0	Less than 20% of body is covered with feces
	1	20 to 50% of body is covered with feces
	2	More than 50% of body is covered with feces
Huddling^1^	0	No pigs showing huddling behavior
	1	Less than 20% of pigs show huddling behavior
	2	More than 20% of pigs show huddling behavior
Panting^1^	0	No pigs are panting
	1	Less than 20% of pigs are panting
	2	More than 20% of pigs are panting
Shivering^1^	0	No pigs are shivering
	1	Less than 20% of pigs are shivering
	2	More than 20% of pigs are shivering
Facility condition^1^	0	No facility damage inside the pen
	2	There is facility damage inside the pen
Wounds on body	0	No wounds on pig body
	2	Wound on any part of pig body
Tail biting	0	No existence of tail biting
	2	Visible fresh blood on the tail and/or evidence of swelling and infection and/or part of the tail missing
Lameness	0	Normal
	2	Severely lame or not able to walk
Pumping	0	No evidence of labored breathing
	2	Evidence of labored breathing
Scouring^1^	0	No liquid manure visible in the pen
	2	Liquid manure visible in the pen
Twisted snouts	0	No evidence of twisted snouts
	2	Evidence of twisted snouts
Hernia	0	No hernia
	2	Hernia/ruptures observed in pig
Rectal prolapse	0	No rectal prolapse
	2	Rectal prolapse observed in pig
Skin condition	0	Normal skin condition
	2	Pig has inflamed, discolored, or spotted skin
Veterinary-client-relationship^3^	0	(1) There is an associated veterinarian that visits the farm regularly to check animal health conditions. (2) The veterinarian is readily available to follow up when health problems occur on the farm.
	1	Only one aspect of previous point is fulfilled
	2	None of the previous points are fulfilled
Medication record^3^	0	Medication record exists
	2	Medication record does not exist
Euthanasia methods^3^	0	1. There is capable person with sufficient knowledge to euthanize pigs. 2. The method used is safe for human and animal.
	1	Only one aspect of previous point is fulfilled
	2	None of the previous point is fulfilled

^1^Pen level.

^2^House level.

^3^Farm level.

Pigs were individually analyzed for their body condition, bursitis, manure on body, lameness, wounds on body, tail biting, pumping, twisted snouts, rectal prolapse, skin condition, ruptures, and hernia. Huddling, shivering, panting, feed quality, facility condition, and scouring were analyzed at the pen level (Table 2). Huddling, shivering, panting, coughing, and sneezing were observed outside the pens. All other measures were assessed inside the pen in order to better observe the pig body. Manure on the body, skin condition, bursitis, and wounds on body were assessed only on one side of each pig, as a previous study by Courboulay and Foubert [7] showed that there was no statistical difference between the left and right side of the animal body for these observations.

**Figure 1:**
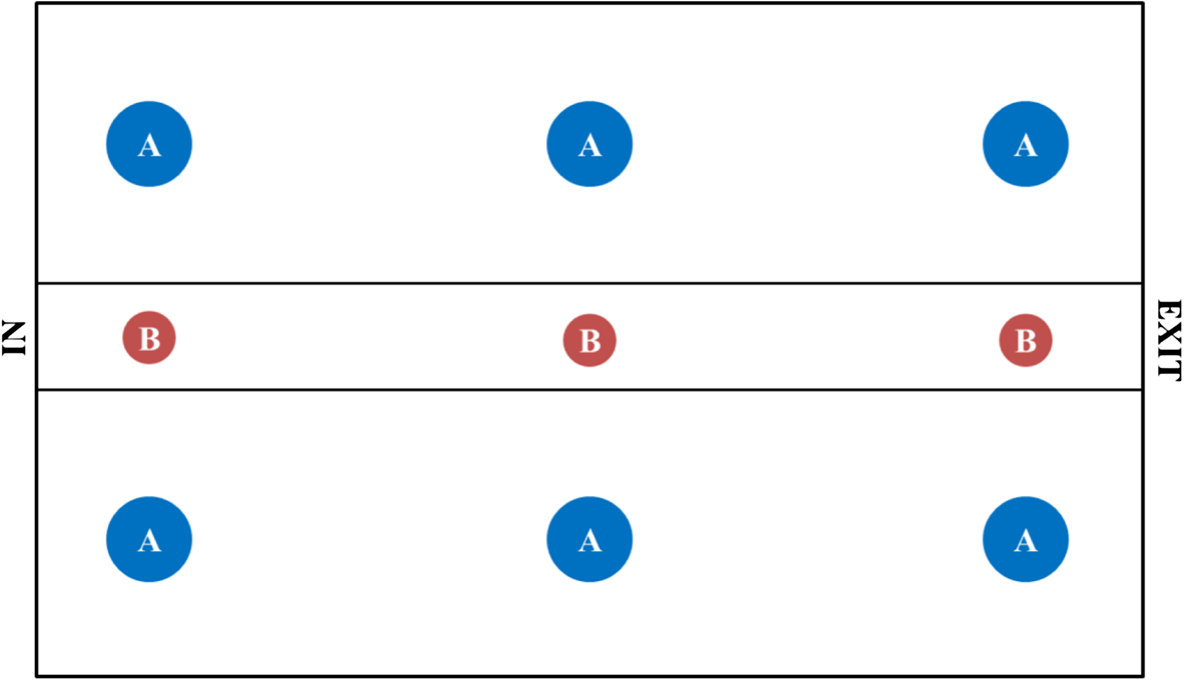
**Sampling points for the environment-based assessment.** (A) Sampling point for ammonia concentration and microclimate parameter; and (B) Sampling points for particulate matter concentration.

Environment-based measures such as ammonia concentration, temperature, and relative humidity were determined from six points inside the pig house. The sampling points were located inside the pen at the pig nose and body height for ammonia concentration and microclimate parameters (temperature and relative humidity), respectively [8]. Particulate matter concentration was determined from three points of the aisle, as it would be difficult to keep the instrument safe from the pig if the measurement was done inside the pen. Details on the sampling points are shown in Figure 1. Ammonia concentration was measured on site using a detector tube (No. 3 L, GASTEC, Kanagawa, Japan). Indoor air was sampled using a gas sampling pump kit (Model GV-100S, GASTEC, Kanagawa, Japan). Microclimate parameters (temperature and relative humidity) were measured using a Climomaster device (Model A531, KANOMAX, Osaka, Japan). Particulate matter was measured using an aerosol mass monitor (Model GT-331, SIBATA, Soca-city, Japan). Particulate matters that were analyzed included PM_10_ (average aerodynamic diameter ≤ 10 μm), PM_7_ (average aerodynamic diameter ≤ 7 μm), PM_2.5_ (average aerodynamic diameter ≤ 2.5 μm), PM_1_ (average aerodynamic diameter ≤ 1 μm), and total suspended particles (TSP) [9].

### Measurement of appropriate behavior

Behavior measurement was assessed based on Welfare Quality® Assessment Protocols for growing pigs [5]. Behavior observation included social behavior, exploratory behavior, human-animal relationship, and qualitative behavior assessment.

Social behavior and exploratory behavior were measured five times using scan samplings with 2.5 minute intervals between each scan conducted at three observation points [5]. Each observation point consisted of 20–40 pigs for a total of 60–120 pigs. Exploratory behavior was measured when enrichment material was available in the house and showed that almost all pig houses in Republic of Korea do not use any bedding or enrichment material.

Good human-animal relationship was measured by observing fear of humans. Fear of humans was assessed by entering the pens, walking around the group slowly until returning to the starting point, and then waiting for 30 s. Then surveyor walked around slowly again in the opposite direction. The response was scored as 0 or 2. Zero means that up to 60% of pigs panicked, whereas 2 means more than 60% of pigs showed panic responses [5]. Qualitative behavior assessment was observed at two points for each pig house. The duration of each observation was 10 minutes for a total of 20 minutes for each pig house. There are 20 descriptions of behavior (active, relaxed, fearful, agitated, calm, content, happy, tense, enjoying, frustrated, sociable, bored, playful, distressed, positively occupied, listless, lively, indifferent, irritable, and aimless) that were observed on a minimum to maximum scale. A maximum score means that the behavior was dominant, whereas a minimum score means that there was an absence of the behavior in the observed animals. The total length of the scale is 125 mm long.

## Results and discussion

### General farm information

Farm A reared about 152 pigs that were divided into 15 different pens. For farm A, average pig age and weight were 9 weeks and 24 kg, respectively. Pigs in farm A were in early growing phase, which explains their lower body weight. The pen area was approximately 3.64 m^2^ with a feeder area of approximately 0.24 m^2^. This indicates that the available area for pigs was approximately 3.4 m^2^ in each pen. Farm B reared about 138 pigs that were divided into 30 different pens. For farm B, average pig age and weight were 18 weeks and 80 kg, respectively. Pigs in farm B were in early fattening phase, which explains their medium body weight. The pen area was approximately 4.29 m^2^ with a feeder area of approximately of 0.37 m^2^. This indicates that the available area for pigs was approximately 3.92 m^2^ in each pen. Space allowances in farms A and B were approximately 0.336 and 0.853 m^2^ per pig, respectively. Pigs in both farms were fed manually once a day. Moreover, cleaning in both farms was done once before pigs were housed.

### Measurement of good feeding, good housing, and good health

In intensive farming systems, prevalence of poor body condition (score 2) is very low since pigs are usually fed *ad libitum* [10]. This often results in a low assessment sensitivity of body condition when using Welfare Quality® Assessment Protocol. Welfare Quality® Assessment Protocol uses a binary scoring system (0 or 2) for body condition, which means it can only differentiate between a very poor body condition and good body condition. Therefore, another scoring method is necessary. The current welfare assessment protocol has a score of 0, 1, or 2 to measure pig body condition, thus allowing easier distinction of good, moderate, or poor body condition, respectively. A new criterion (feed quality) was also introduced for the good feeding principle in the developed welfare assessment protocol. Feed quality analysis was proposed since the climate of Republic of Korea is humid, especially in the summer, which means feed can easily rot. Having a feed condition score provides information on whether or not feed is rotten, which would affect the pig digestive system and result in watery feces (diarrhea).

**Figure 2:**
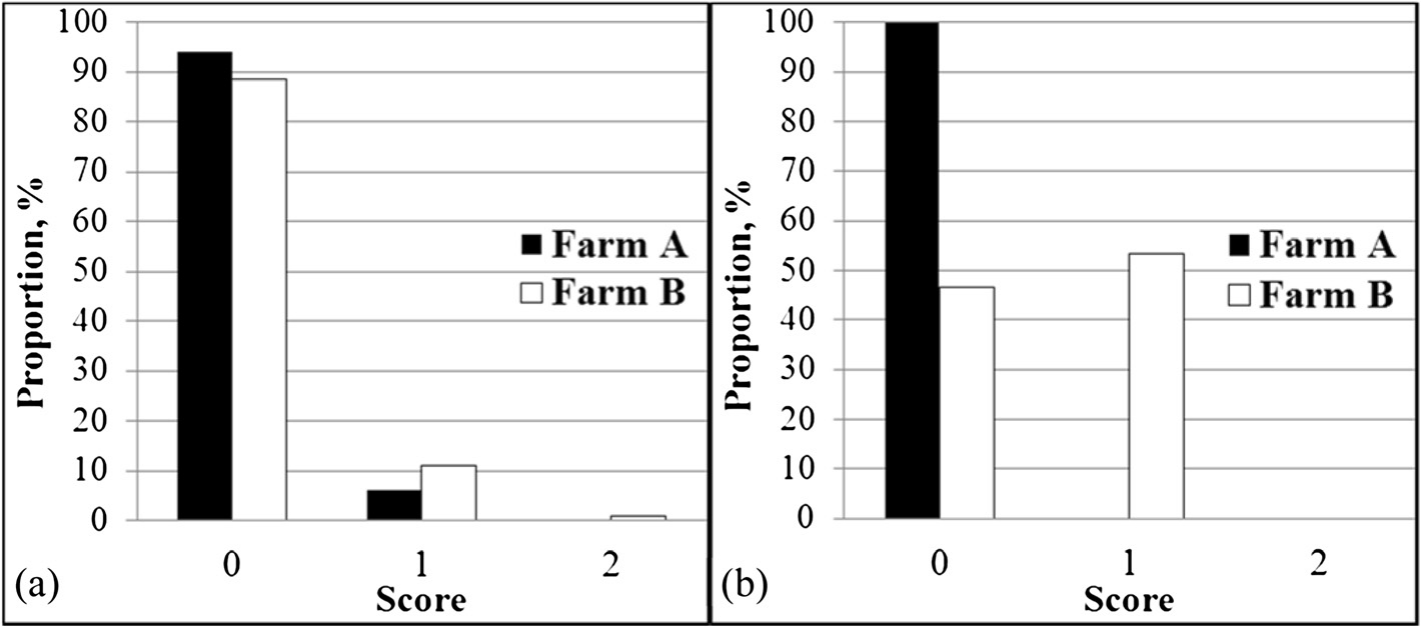
**Validation result of developed welfare assessment protocols in good feeding principles. (a)** Body condition and **(b)** Feed quality.

In terms of body condition, assessment of the two experimental farms showed a moderate body condition (score 1) (Figure 2a). Proportions of pigs showing a moderate body condition in farms A and B were 6.0% and 10.87%, respectively. This result shows that scoring for moderate body condition (scoring 1) is a necessary criterion in the welfare assessment protocol. For feed quality, good feed quality (score 0) and moderate feed quality (score 1) were observed in farm B, whereas only good feed quality (score 0) was observed in farm A (Figure 2b). Proportions of pens showing good and moderate feed quality in farm B were 46.67% and 63.33%, respectively. The moderate feed quality observed in farm B might be due to high relative humidity, which ranged from 62.6 to 93.8% with an average of 79.30% (Table 3). The relative humidity in farm A ranged from 65.9 to 70.05% with an average of 67.69%. The lower relative humidity in farm A could explain the lack of moderate or poor feed quality. This result suggests that feed quality could be a problem in intensive farming systems, especially for farms located in humid areas. Therefore, feed quality is a necessary criterion in the welfare assessment protocol.

**Table 3:** **Microclimate parameter and particulate matter concentration in farm A and farm B during validation of the developed welfare assessment protocol**

**Parameters**	**Farm A**	**Farm B**
Temperature,°C	28.52 ± 0.38	27.35 ± 0.86
Relative Humidity, %	67.69 ± 1.37	79.3 ± 10.2
NH_3_, ppm	2.83 ± 2.21	6.35 ± 3.23
PM_10_, μg/m^3^	129.1 ± 18.71	110.13 ± 20.53
PM_7_, μg/m^3^	88.9 ± 16.94	73.77 ± 22.98
PM_2.5_, μg/m^3^	38.97 ± 3.32	36.17 ± 10.07
PM_1_, μg/m^3^	22.33 ± 3.13	20.93 ± 5.86
TSP_,_ μg/m^3^	273.6 ± 92.77	287.50 ± 64.52

Ammonia (NH_3_) is a highly irritating, colorless gas that is produced by microbial degradation of pig urine and the nitrogenous compound fraction of feces, and it is representative of gaseous compounds in pig houses. Moreover, NH_3_ accumulation inside pig houses is an indicator of ventilation failure. Ammonia was chosen as the determined factor since it can be analyzed on-site. The National Pork Board US [6] suggested that the NH_3_ concentration should not exceed 50 ppm. In our results, both pig houses had NH_3_ concentrations lower than 50 ppm. The average NH_3_ concentration in pig houses A and B were 2.83 and 6.35 ppm, respectively (Table 3). This result indicates that the ventilation systems were adequate in both houses. Although the NH_3_ concentration during validation of the developed welfare assessment protocol was quite low, NH_3_ concentration is still a necessary criterion for welfare assessment since high NH_3_ levels are known to reduce pig performance (average weight gain and feed efficiency), health, and welfare status [11-13].

Particulate matter (PM) is one of the primary air pollutants in livestock housing and almost completely consists of organic and biological matters derived from feed, skin, livestock hair, bedding material, urine, feces, and microorganisms. PM has several adverse effects, such as affecting health of livestock and transporting infectious diseases (microorganisms and toxic compounds) in pig houses [14,15]. Although there is no threshold for PM concentration inside pig houses, addition of PM as a criterion of welfare assessment needs to be considered since PM concentration is related to respiratory diseases such as lung inflammation, irritation of the respiratory system, and rhinitis. It is known that a PM concentration higher than 3700 μg/m^3^ increases mortality and pneumonia or pleuritis prevalence in fattening pigs [16,17]. Several PM sizes were analyzed since any PM larger than 10 μm is deposited in the nasal passage, PM between 5 to 10 μm is deposited in the upper respiratory tract, and PM smaller than 5 μm (respirable dust) is deposited in the lower respiratory tract and lungs [14,18]. Thus, each PM size might have different effects on the pig body, which means it is necessary to measure PM sizes in pig houses. The PM concentration at both farms was relatively low (Table 3), indicating no welfare problem related to PM concentration.

**Figure 3:**
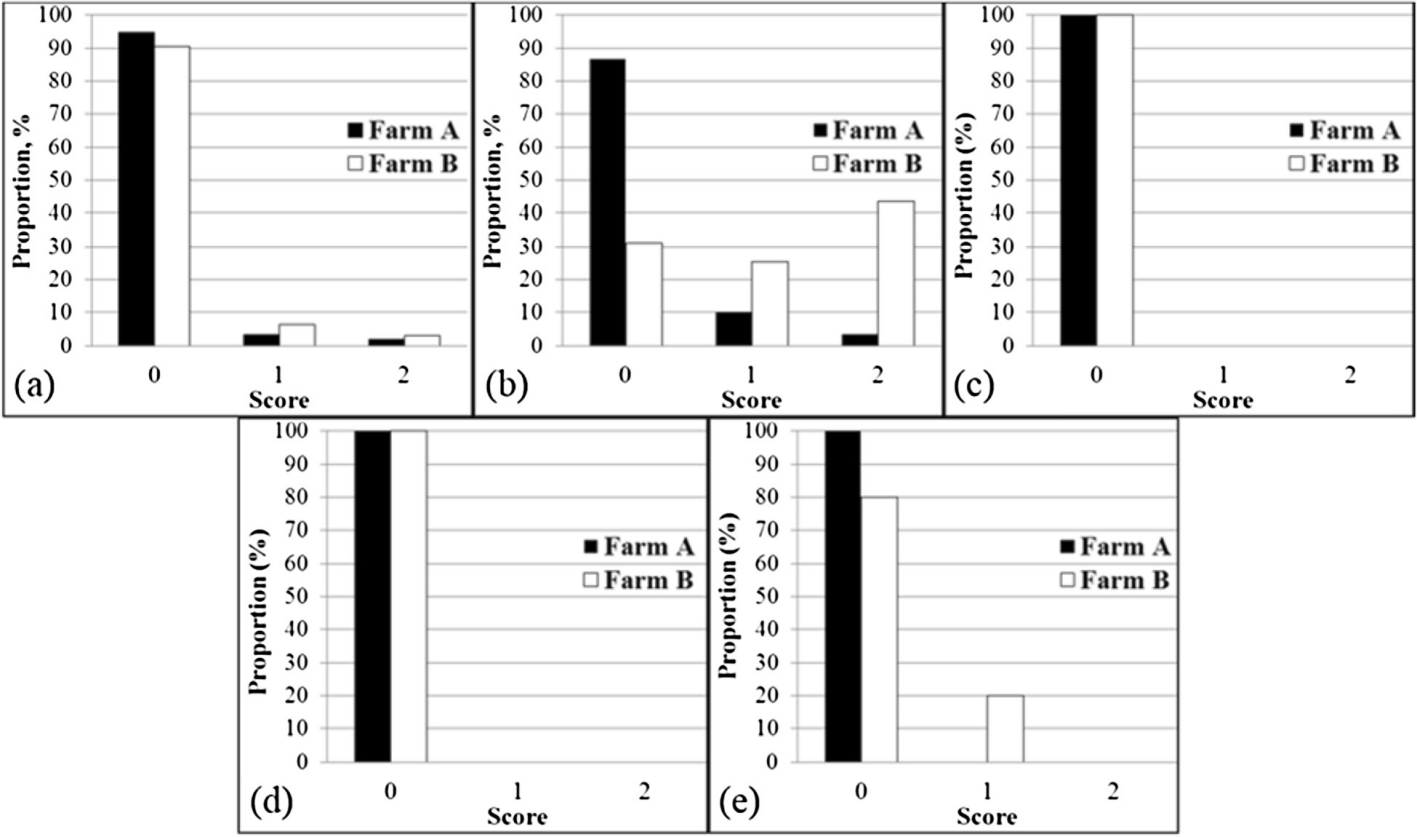
**Validation results of developed welfare assessment protocol criteria following good housing principles. (a)** Bursitis; **(b)** Manure on the body; **(c)** Huddling; **(d)** Shivering; and **(e)** Panting.

Bursitis and manure on the body indicates comfort during rest and thus becomes an important factor for assessing pig welfare status [19,20]. In terms of bursitis, most pigs (more than 90%) showed no signs of bursitis (score 0), indicating good welfare quality in both farms. Nevertheless, medium bursitis (score 1) and severe bursitis (score 2) were more common in farm B than farm A (Figure 3a). One explanation is that pig body weight was higher in farm B compared to farm A. Higher body weight creates more tension on the leg and in turn results in bursitis. In terms of manure on the body (Figure 3b), farm A showed good welfare quality, as more than 86% of pigs had no or little manure on their bodies (score 0). However, the welfare criteria in terms of manure on the body must be improved since more than 10% of pigs had a medium amount of manure on their bodies (score 1). On the other hand, farm B showed poor welfare quality, as more than 60% of pigs had a medium or large amount of manure on their bodies (scores 1 and 2). This may be due to a higher room temperature in farm B (27.4°C) than the recommended temperature of growing pigs in the weight range from 68 to 100 kg, which is around 10 to 24°C [21]. High temperature induces wallowing behavior in pigs, which reduces body temperature. In farm B, the welfare of pigs in terms of manure body condition can be improved by reducing the room temperature and increasing cleaning frequency.

Huddling, shivering, and panting are animal-based indicators of thermal comfort [10]. In terms of huddling (Figure 3c) and shivering (Figure 3d), both farms showed good welfare quality based on the lack of huddling or shivering behavior in pigs (score 0). However, about 20% of pens in farm B indicated panting behavior (Figure 3e), which is one way for pigs to dissipate body heat. As explained previously, the room temperature in farm B (27.4°C) was higher than the recommended temperature for growing pigs in the weight range from 68 to 100 kg.

Physical health refers to the state of an animal’s brain and body in an attempt to cope with pathology. Therefore, health is an important aspect of animal welfare and must be appropriately considered [22,23]. In terms of absence of injury, the following scores were measured: lameness, wounds, and tail biting. There was no evidence of injuries in farm A, whereas around 2.2% of pigs had wounds in farm B (Table 4). The warning threshold for wounds in the protocol is 5%, which means that pigs in farm B can be considered as normal. However, lameness measurement could not be performed properly due to insufficient space, which made it almost impossible for the pig to walk. Additionally, low light intensity made it difficult to do the observation.

**Table 4:** **Validation results of developed welfare assessment protocol following good health principles**

**Parameters**	**Farm A**		**Farm B**	
	**0**	**2**	**0**	**2**
Lameness, %	100	0	100	0
Wounds on body, %	100	0	97.8	2.2
Tail biting, %	100	0	100	0
Pumping, %	91.9	0.9	100	0
Twisted snouts, %	100	0	100	0
Rectal prolapse, %	100	0	100	0
Scouring, %	40	60	100	0
Skin condition, %	100	0	100	0
Ruptures and hernia, %	100	0	100	0
Abscesses, %	100	0	100	0

Almost all of the pigs experienced tail docking, which made it difficult to differentiate whether or not tail biting had occurred. Tail docking is considered as an effective method of reducing tail biting, although recent studies have shown it to have many disadvantages such as pain sensitization, risk of infection, and ethical considerations [5]. In addition, our category defined tail biting as visible fresh blood on the tail, evidence of swelling and infection, or part of the tail missing. In both farms, most pigs showed reduced tail lengths due to tail docking, so we did not count those cases as tail biting.

In term of diseases, the following scores were measured: mortality rate, coughing, sneezing, pumping, twisted snouts, rectal prolapse, scouring, skin condition, ruptures, and hernias and abscesses. In farm A, only one pig (0.9%) showed evidence of pumping (Table 4), which does not exceed the warning threshold (1.8%). In addition, there was evidence of scouring in 60% of total pens (9/15) in farm A, and the alarm threshold for scouring in our protocol is 15%. Additionally, pens were classified as either with liquid feces or without liquid feces. This classification is not sufficient to differentiate the cause of scouring. To exactly determine the problem, detailed information about factors such as fecal consistency and color are needed. Nevertheless, the presence of scouring only in farm A might be due to the young age of the pigs (about 9-weeks-old), which is an age prone to scouring. Environmental changes in early growing pigs can cause a stress response that in turn affects the incidence and severity of enteric disease [24]. The mortality rates in both farms were low (0.05% in farm A and 0.2% in farm B) with a warning threshold of 2.6%. This result shows that the welfare status of the farms based on mortality rate was good.

Several studies have concluded that lung inflammation can be estimated by calculating frequency of coughing [5]. In farm A, coughing frequency was 0.31 with a warning threshold of 0.15. Therefore, the causes behind coughing should be determined. In farm A (0.04), sneezing was under the threshold value of 0.27. In farm B, both coughing (0.01) and sneezing (0.08) were maintained under threshold values. Therefore, there was no indication of a health problem in farm B.

In terms of health management, the following scores were measured: veterinarian-client-patient relationship and medication records. Both farms had medication records, but they did not confirm any veterinarian-client-patient relationship. Our measurement included two possible situations: (1) The veterinarian has recently seen and is personally acquainted with the keeping and care of the animal(s) via medically appropriate and timely visits to the premises where the animal(s) are kept; (2) The practicing veterinarian is readily available for follow-up in case of adverse reactions or failure of the therapy regimen. Neither of these was fulfilled in either farm. Good health management practices, which include a good veterinarian relationship, is important for maintaining good herd health [6]. Therefore, this parameter can be added to assess the welfare status of pig farms.

In terms of euthanasia, the following scores were measured: euthanized animals and euthanasia methods. Using the protocol, euthanized animals were calculated by comparing the amount of euthanized animals to total deaths. Euthanasia is used only when the animal is suffering a condition that either cannot be cured or is uneconomical to be cured. Further, there are situations in which immediate euthanasia is required as a response to inadequate animal conditions. Therefore, appropriate and timely euthanasia methods are critical for pig welfare [6]. However, in both farms, euthanasia practices were not used. Immediate action should be undertaken to improve animal welfare related to this area. Lastly, both farms had a hospital pen. Further information on the use of these hospital pens could be useful since this protocol only determined whether or not one was available.

### Measurement of appropriate behavior

Appropriate behavior assessments focused on social behavior, human-animal relationships, and qualitative behavior. Exploratory behavior was not assessed since there was no enrichment material provided for the growing pigs in the visited farms. A lack of enrichment material such as straw can have many effects. From a behavioral point of view, this can reduce the incidence of behavioral expression in pigs [25]. This absence of any enrichment material is most likely due to a lack of knowledge on behalf of the farmer that an enrichment material increases mental health of pigs or too high production costs.

Social behavior percentage was calculated based on total active behavior, whereas resting behavior was not considered. As shown in Table 5, there were differences in the percentage of social behavior between farms A and B, even though it was not significant. The percentage of negative social behavior was more pronounced in farm A, most likely due to a higher number of animals per pen. The space allowance in farms A and B were 0.336 m^2^ and 0.853 m^2^ per pig, respectively. It is known that the aggression potential is affected by crowding and that limited space causes competition for resources. Social behavior is also affected by environmental factors as well as management of the farm [1]. Limited access to resources such as food and water precipitates a competitive situation that leads to aggression, which is considered as negative social behavior. Intensive farming systems are characterized by elevated intensity and frequency of negative social behavior [20]. Based on our observations, space allowance has a strong effect on observed social behavior, so this information should always be recorded as a measurement of animal welfare. However, a high percentage of positive social behavior does not necessarily imply a good situation, as positive interactions sometimes result in negative ones. Temple et al. [20] showed that there is a high correlation between negative and positive social behaviors.

**Table 5:** **Animal behavior recorded during observation**

**Behavior**	**Farm A**	**Farm B**
Active behavior, %	56.24	24.41
Other, %	79.42	76.89
Social behavior, %	20.57	23.61
Negative social behavior, %	32.81	5.88
Positive social behavior, %	67.19	94.12
Pen with score 2 (show panic response), %	46.67	30

The human-animal relationship (HAR) is important and influences both animal production and welfare [26]. Therefore, this criterion must be measured to assess animal welfare status. Fear of humans is measured to assess welfare since sudden and prolonged fear may severely harm animal welfare and have negative consequences on productivity and product quality [26]. In this study, the HAR in the form of a fear-of-human score was expressed as the percentage of pens with more than 60% of pigs showing a panic response. As shown in Table 5, farms A and B showed scores of 46.67% and 30%, respectively. Fear of humans is a direct reflection of how the pigs are handled. There are also several factors that influence fear of humans, including age, genetic background, and space allowance [10]. The age of growing pigs was 9 weeks in farm A and 18 weeks in farm B. Moreover, space allowances in farms A and B were 0.336 m^2^/pig and 0.853 m^2^/pig, respectively. These differences in age and space allowance per pen between the two farms might explain many of the factors above.

Results of the qualitative behavior assessment are expressed on a millimeter scale ranging from 0 to 125 mm. Zero is the minimum on the qualitative behavior scale while 125 is the maximum. The results of this assessment reveal high variability (Table 6), possibly due to subjectivity of measurement. Enriched environmental conditions can result in different behavioral expression in animals [27]. Therefore, measurement of behavioral expression plays an important part in measuring animal welfare. Even though it is difficult to interpret these results, this assessment provides information on animal-based welfare based on their emotional state. Our results also suggest that behavior measurement is needed to properly assess and improve animal welfare.

**Table 6:** **Qualitative behavior assessment observation scale expressed in millimeters**

**Description**	**Scale (mm)**	
	**Mean**	**SD**
Active	54.5	40.31
Relaxed	73	29.7
Fearful	12.5	17.68
Agitated	1	1.41
Calm	79	45.25
Content	79	2.83
Happy	24.5	17.68
Tense	12	16.97
Enjoying	21.5	7.78
Frustrated	13	18.38
Sociable	49.5	37.48
Bored	60.5	27.58
Playful	30.5	14.85
Distressed	14	19.8
Positively occupied	34	14.14
Listless	1.5	2.12
Lively	54	25.46
Indifferent	49.5	48.79
Irritable	15.5	21.92
Aimless	73	31.11

## Conclusions

This study is a first step in developing a new pig welfare assessment protocol that combines animal-, environment-, and management-based measures. Environment- and management-based measures can help to assess welfare status whenever animal-based measures are difficult. This study also provides an explanation of each criterion in the developed pig welfare assessment protocol.

Assessment showed that some farms have moderate (score 1) or poor feed quality (score 2), especially those farms located in a relatively high humidity region. Specifically, in farm B, 63.33% of pens showed moderate feed quality. Modification of body condition score into a 3-point scale showed that pigs could be divided into three classes: good body condition (score 0), moderate body condition (score 1), and poor body condition (score 2). The prevalence of pigs with moderate body condition in farms A and B were 6.0% and 10.87%, respectively.

The validation results of the two farms show that the developed protocol could be utilized to assess welfare status in an intensive pig farming system. Further improvement of the developed protocol is needed, either by eliminating one or several measurement criteria or by changing the scoring system.
